# A case report of a child with pulmonary hypertension associated with SARS-CoV-2 infection

**DOI:** 10.3389/fped.2024.1336589

**Published:** 2024-02-08

**Authors:** Kentaro Okunushi, Hironobu Kobayashi, Yuri Yoh, Masaya Kunimatsu, Tadashi Shiohama, Tomozumi Takatani, Hiromichi Hamada

**Affiliations:** Department of Pediatrics, Graduate School of Medicine, Chiba University, Chiba, Japan

**Keywords:** SARS-CoV-2, pulmonary hypertension, children, panhypopituitarism, growth hormone, portal hypertension

## Abstract

We encountered a pediatric case of pulmonary hypertension triggered by severe acute respiratory syndrome coronavirus 2 (SARS-CoV-2) infection. A 14-year-old girl was brought to the emergency department of our hospital with fever, respiratory distress, and impaired consciousness. She tested positive for SARS-CoV-2 upon a polymerase chain reaction examination and had prolonged hypoxemia without pneumonia. An echocardiography revealed elevated right ventricular pressure. She was diagnosed with pilocytic astrocytoma at the age of 10 years and underwent a resection of a pituitary tumor. Hormone replacement therapy was administered postoperatively, but her growth hormones were not activated because of concerns about tumor recurrence. Echocardiography at the age of 13 years showed normal right ventricular pressure. On admission, she had an abnormal liver function, elevated liver fibrosis markers, a decreased platelet count, and hepatosplenomegaly, suggesting pulmonary and portal hypertension. The diagnosis was pulmonary hypertension associated with SARS-CoV-2 infection. The mechanism of the pulmonary hypertension was thought to be portal hypertension owing to growth hormone deficiency and SARS-CoV-2 infection. The patient's symptoms improved with oxygenation and bed rest without additional targeted pulmonary hypertension therapy, and her right ventricular pressure decreased. This case demonstrates that a pediatric patient with subclinical pulmonary hypertension may develop pulmonary hypertension triggered by SARS-CoV-2 infection.

## Introduction

Severe acute respiratory syndrome coronavirus 2 (SARS-CoV-2) infection has been the subject of a considerable number of clinical reports, and a detailed picture of the disease has emerged, especially in the acute phase. The pathogenesis of SARS-CoV-2 infection in children is also becoming better understood. The clinical course has been reported to be milder in children than in the elderly; however, there have been deaths in children, the majority of which were caused by neurological and cardiovascular complications (1, 2).

There have been reports of pulmonary hypertension as a complication of SARS-CoV-2 infection, but most have concerned adult cases (3). This report describes the case of a pediatric patient who was admitted to hospital in the acute phase of SARS-CoV-2 infection with fever, respiratory distress, and impaired consciousness and was diagnosed as having pulmonary hypertension as a complication of SARS-CoV-2 infection.

## Case description

The patient was a 14-year-old girl who was brought to the emergency department with fever, respiratory distress, and impaired consciousness. She developed fever on the first day of illness and became aware of respiratory distress while walking on the second day. Later in the day, she was observed to have episodes of loss of consciousness and was taken to the emergency department of another hospital. On arrival, she had a Glasgow Coma Scale score of E2V3M5, SpO_2_ of 92%, and blood glucose level of 41 mg/dL. Her level of consciousness gradually improved with the administration of hydrocortisone and glucose, but she continued to have respiratory distress and hypoxemia. An echocardiography performed on day 3 of illness revealed elevated right ventricular pressure, and the patient was transferred to our hospital.

At the age of 10 years, she was diagnosed with pilocytic astrocytoma and underwent an endoscopic transnasal resection of a pituitary tumor. She was subsequently diagnosed with panhypopituitarism and was recommended levothyroxine, hydrocortisone, and desmopressin acetate hydrate. At the age of 13 years, a physical examination at her school led to a diagnosis of long QT syndrome. An echocardiography at that time showed normal right ventricular pressure. She had no history of syncope.

The patient had a height of 143.8 cm (−2.4 SD), weight of 43.0 kg (−0.9 SD), body mass index of 20.8, temperature of 36.7°C, heart rate of 83 beats/min, blood pressure of 149/109 mmHg, respiratory rate of 26 breaths/min, oxygen saturation of 90%–91% on room air, and oxygen saturation of 97% on oxygen at 1 L/min. She was conscious but had orthopnea and was using her shoulder muscles for accessory breathing. The color of her skin was good. Lung sounds were clear, and respiratory sounds were not diminished. Her heart rhythm was regular with a mild hypertonicity of tone II. Tone III was inaudible, and no heart murmur was noted. The abdomen was flat and soft with no hepatosplenomegaly. There was no peripheral coldness of the extremities, but mild edema was noted.

**Figure 1:**
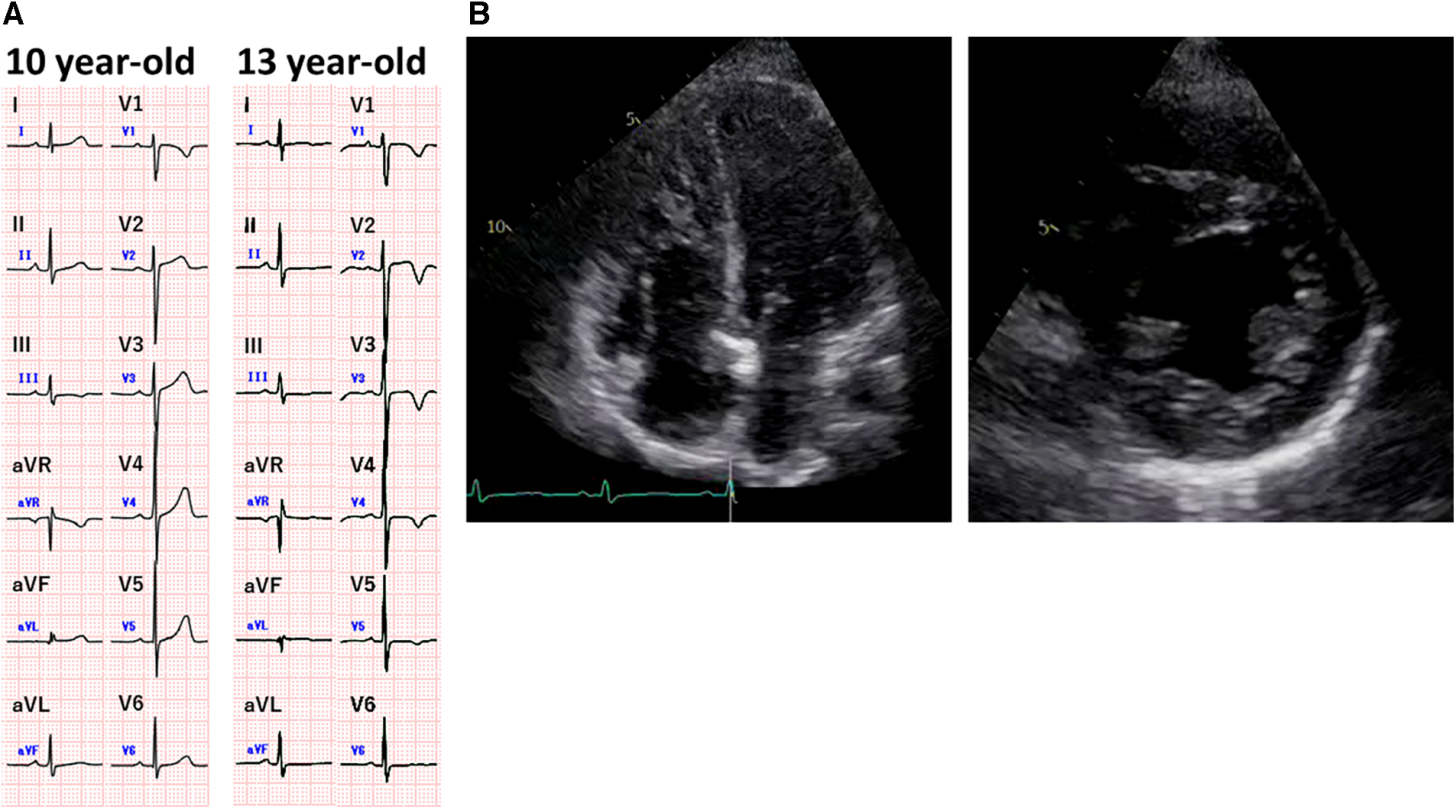
Cardiac screening data before the onset of severe acute respiratory syndrome coronavirus 2 infection. (**A**) Electrocardiograms recorded at the age of 10 years before surgery for a brain tumor and at the age of 13 years at the time of a physical examination in junior high school. The electrocardiogram obtained at the age of 13 years shows negative T waves in leads V1–V5. The QTc interval was 433 ms. (**B**) Echocardiographic images obtained at the age of 13 years after abnormal electrocardiographic findings. There is no evidence of elevated right ventricular pressure in either the four-chamber view or the short-axis view.

**Figure 2:**
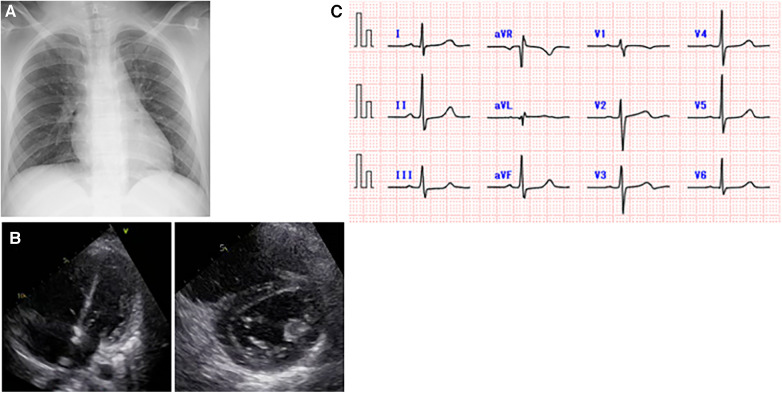
Cardiac examination data after the onset of severe acute respiratory syndrome coronavirus 2 infection. (**A**) A chest x-ray on day 2 of illness. There was no cardiomegaly or pulmonary congestion. There were no obvious radiographic findings suggestive of pneumonia that would explain the hypoxia. (**B**) Echocardiographic images on day 3 of illness. Elevated right ventricular pressure was found in the four-chamber and short-axis views, with an estimated tricuspid regurgitation pressure gradient of 64 mmHg and a maximum estimated pulmonary valve regurgitation pressure gradient of 47 mmHg. (**C**) An electrocardiogram obtained on day 3 of illness showed no abnormal T waves.

A chest x-ray showed no cardiomegaly. A chest computed tomography scan showed no abnormalities in the lung fields or pulmonary vessels. An echocardiography revealed an enlargement of the right atrium and ventricle, as well as elevated right ventricular pressure, with an estimated tricuspid regurgitation pressure gradient (TRPG) of 64 mmHg and an estimated maximum pulmonary valve regurgitation pressure gradient of 47 mmHg (Figure 1). The electrocardiography did not show pulmonary P waves or right ventricular hypertrophy (Figure 1). Negative T waves were observed in leads V1–V5 at the age of 13 years after a resection of a pituitary tumor (Figure 2), but they were not detected on the electrocardiogram recorded during the current admission at 14 years of age. The corrected QT time was 0.331 s. Blood tests revealed thrombocytopenia and elevated fibrin–fibrinogen degradation, aspartate aminotransferase, and B-type natriuretic peptide levels. Elevated total bile acids and liver fibrosis markers (i.e., hyaluronic acid and type 4 collagen) were also found. She tested negative for all autoantibodies examined for collagen disease. However, the result of a polymerase chain reaction test for SARS-CoV-2 was positive (Table 1).

**Table 1:** Blood tests performed on day 2 of illness.

		Reference value			Reference value
WBC	3,200 /μL	(3,300–8,600)	aDNA ab	(−)	
RBC	422·10^4 ^/μL	(386–492)	aSS-A ab	(−)	
Hb	11.1 g/dL	(11.6–14.8)	aSS-B ab	(−)	
PLT	9.1·10^4 ^/μL	(15.8–34.8)	aRNP ab	(−)	
PT-INR	1.01	(0.8–1.2)	aSm ab	(−)	
APTT	36.6 s	(24.3–36.0)	aScl-70 ab	(−)	
FIB	267 mg/dL	(150–400)	MPO-ANCA	(−)	
FDP	10.9 μg/mL	(<5)	PR3-ANCA	(−)	
AST	55 U/L	(13–30)	TBA	32.2 μmol/L	(<10)
ALT	29 U/L	(7–23)	NH_3_	54 μg/dL	(12–66)
LDH	280 U/L	(124–222)	HBs Ag	(−)	
ALP	151 U/L	(60–390)	HCV ab	(−)	
γ-GTP	38 U/L	(9–32)	Cu	100 μg/dL	(68–128)
ChE	393 U/L	(201–421)	Hyaluronic acid	54 ng/mL	(<50)
T-Bil	0.5 mg/dL	(0.4–1.5)	Type-Ⅳ collagen 7S	11.2 ng/mL	(<6)
TC	167 mg/dL	(142–248)			
HDL-C	39 mg/dL	(48–103)	COVID-19 PCR	(+)	
LDL-C	95 mg/dL	(65–163)			
CRP	0.53 mg/dL	(<0.14)			
BNP	242pg/dL	(<18.4)			
ANA	(−)				
C3	92.0 mg/dL	(86–160)			
C4	14.1 mg/dL	(17–45)			
CH50	16.2 U/mL	(25–48)			

aDNA ab, anti-DNA antibody; ANA, antinuclear antibody; aRNP ab, antiribonucleoprotein antibody; aScl-70 ab, anti-Scl-70 antibody; aSm ab, antismooth muscle antibody; aSS-A ab, anti-Sjogren Syndrome-A antibodies; aSS-B ab, anti- Sjogren Syndrome-B antibodies; C3, complement component 3; C4, complement component 4; COVID-19, coronavirus disease 2019; Cu, copper; HbsAg, hepatitis B surface antigen; HCV, hepatitis C virus; MPO-ANCA, myeloperoxidase antineutrophil cytoplasmic antibodies; NH3, ammonia; PCR, polymerase chain reaction; PR3-ANCA, proteinase 3 antineutrophil cytoplasmic antibodies; TBA, thiobarbituric acid.

**Figure 3:**
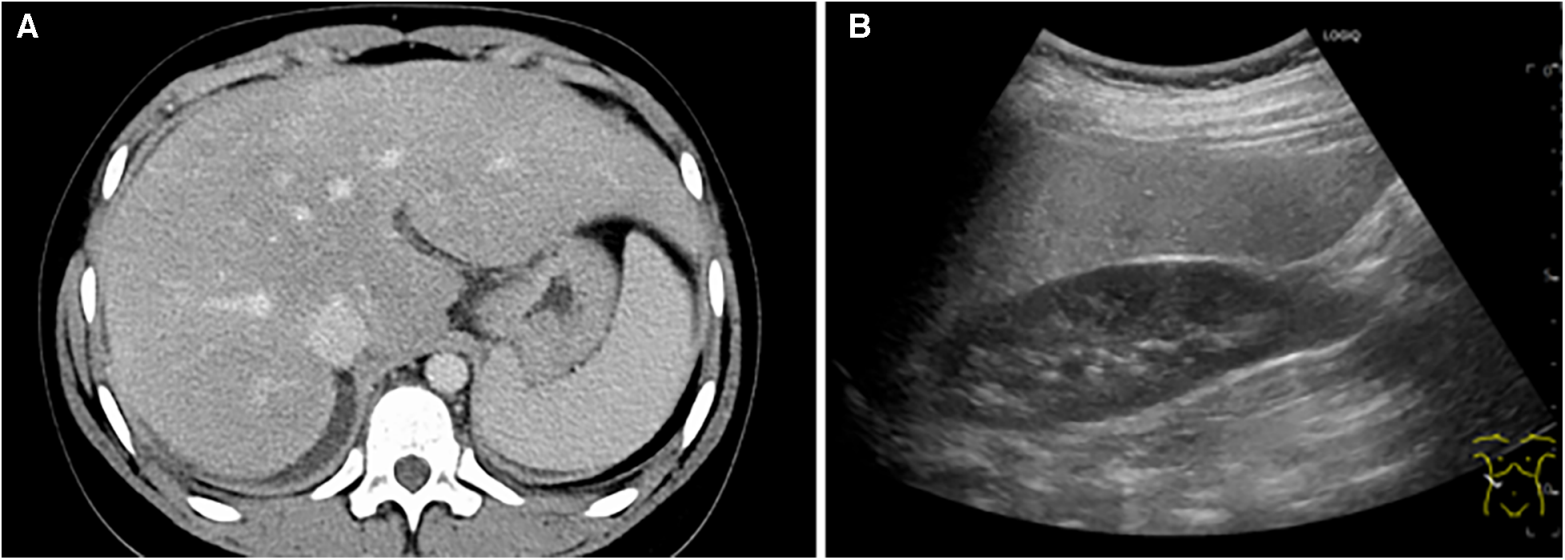
Other imaging data obtained after the onset of severe acute respiratory syndrome coronavirus 2 infection. (**A**) A contrast-enhanced abdominal computed tomography scan showing hepatomegaly and splenomegaly. (**B**) An abdominal echocardiographic image showing increased echoluminance of the liver, which indicates a fatty liver.

Contrast-enhanced computed tomography scans of the abdomen showed hepatosplenomegaly, and an abdominal echocardiography showed a fatty liver (Figure 3). There was no obvious portal vein circulatory shunt.

The above findings indicated SARS-CoV-2 infection, potentially complicated by pulmonary hypertension and liver dysfunction.

## Diagnostic assessment, treatment, and outcome

The patient was diagnosed as having non-alcoholic fatty liver disease and portal hypertension on a background of panhypopituitarism with subclinical pulmonary hypertension caused by the portal hypertension that manifested clinically during SARS-CoV-2 infection.

**Figure 4:**
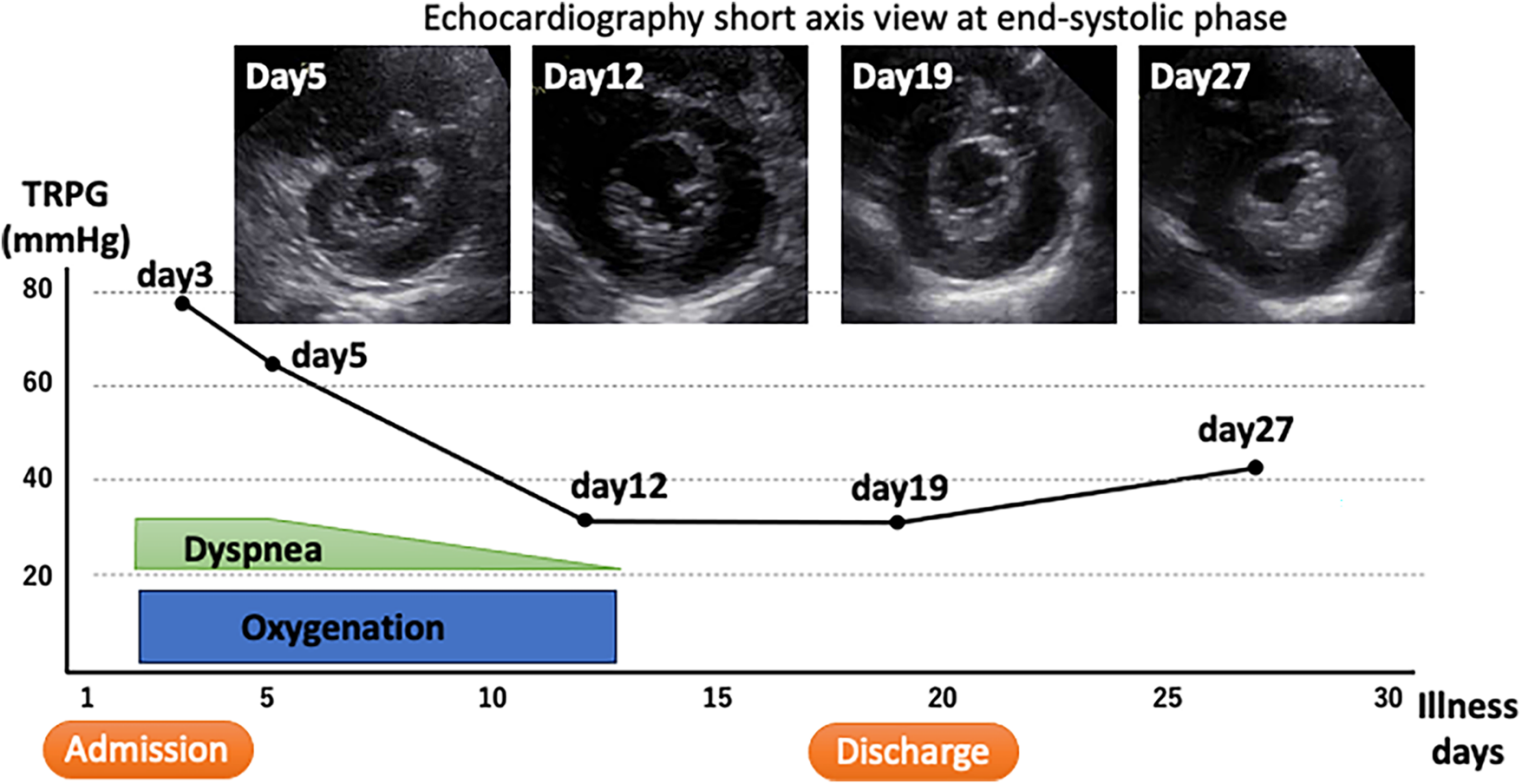
Clinical course. The tricuspid regurgitation pressure gradient, which is a measure of right ventricular pressure, is shown on the vertical axis along with the time course of treatment. The upper image is a short-axis echocardiographic view that is shown with its time course.

The clinical course after admission is shown in Figure 4. Oxygen administration was continued and the patient was placed on bed rest in isolation. She did not require targeted pulmonary hypertension therapy or positive pressure respiratory support. Her complaints of respiratory distress gradually resolved, and an echocardiography on day 12 of illness showed an almost normal ventricular septal morphology and improvement in the TRPG to 31 mmHg. The right ventricular pressure did not increase again after oxygen was stopped and the patient was allowed to mobilize. She was discharged after 19 days in hospital. At the first postdischarge outpatient visit (day 27 after the onset of illness), her TRPG had increased again to 42 mmHg and the ventricular septum appeared to be pushed slightly toward the left ventricle. However, these abnormalities subsequently normalized. Thereafter, the patient has shown no further symptoms and has returned to her normal everyday life.

## Discussion

There have been some previous reports of pulmonary hypertension in pediatric patients with SARS-CoV-2 infection (4–6). Olfe et al. reported a 16-old-year patient with pulmonary hypertension and underlying mitral valve disease (4). Morales-Demori et al. reported on the status of COVID-19 morbidity in a pediatric pulmonary hypertension center (6). They found that of 23 children with COVID-19, 8 were hospitalized and that 3 of these patients required an intensification of targeted pulmonary hypertension therapy. These reports suggest that COVID-19 can lead to overt pulmonary hypertension in children with subclinical pulmonary hypertension. We consider that the pulmonary hypertension in our patient was a complication caused by a combination of postoperative hypopituitarism and SARS-CoV-2 infection.

An electrocardiogram recorded when the patient was 13 years old (1 year before SARS-CoV-2 infection) showed flat T waves in leads II, III, and aVf, negative T waves in leads V1–V5, and flat T waves in V6, all of which were abnormal findings. An abnormal electrocardiogram is often associated with intracranial abnormalities, possibly because of increased catecholamines in response to a hyperactive sympathetic nervous system (7). At the age of 13 years, she had an abnormal electrocardiogram during screening at school. A subsequent echocardiography revealed normal right ventricular pressure. Therefore, it is unlikely that she had pulmonary hypertension before the onset of SARS-CoV-2 infection. During the present admission, there were no findings suggestive of right ventricular hypertrophy on the electrocardiogram. The short-axis echocardiographic image and TRPG value also suggested that the patient did not have pulmonary hypertension to the extent that it exceeded her systemic blood pressure.

The growth hormone (GH) and insulin-like growth factor-1 (the secretion of which is stimulated by the GH) are important factors in terms of reducing fat accumulation and fibrosis in the liver. An inadequate secretion of the GH can lead to liver disease, including non-alcoholic fatty liver disease, non-alcoholic steatohepatitis, and even a cirrhosis of the liver (8). Our patient was consuming levothyroxine, hydrocortisone, and desmopressin acetate hydrate for panhypopituitarism after a resection of a pituitary tumor, but she was not under any medication for activating her GHs because of concerns about tumor recurrence. Mildly elevated alanine aminotransferase and elevated liver fibrosis markers suggested liver damage associated with GH deficiency.

Previous reports indicate that portal hypertension is present in approximately 30% of patients with non-alcoholic fatty liver disease, and that even in the absence of significant fibrosis, portal hypertension is correlated with the severity of fatty liver disease (9). Splenomegaly and thrombocytopenia, which indicate hypersplenism, were also observed in our patient. Elevated liver fibrosis markers were also detected. These findings suggest the possibility of portal hypertension.

Although the pathogenesis of pulmonary hypertension associated with portal hypertension has not been well understood, it is thought to involve increased pulmonary vascular shear stress caused by increased cardiac output and inflammatory cytokines and endotoxins that do not pass through the liver (10).

SARS-CoV-2 infection is established when spike proteins on the surface of SARS-CoV-2 bind to angiotensin 2 receptors, which are highly expressed in alveolar epithelial cells and the vascular endothelium. The viral infection is thought to induce pulmonary hypertension in response to diffuse lung injury, thrombosis, and cytokine storm (11). In the case of SARS-CoV-2, the virus is internalized along with angiotensin 2 receptors by endocytosis, resulting in a loss of function of these receptors in the cell membrane and functional impairment; this is thought to cause vasoconstriction and induce pulmonary hypertension by increasing the amount of angiotensin 2 that is not degraded and facilitating its binding to the type 1 angiotensin receptor (12). This pathophysiology could indicate that SARS-CoV-2 infection exacerbates pulmonary hypertension, although symptoms of pneumonia were not prominent in our patient.

In conclusion, we described the case of a pediatric patient with pulmonary hypertension as a complication of SARS-CoV-2 infection. We consider that she had a pre-existing subclinical pulmonary hypertensive condition as a result of underlying disease that manifested clinically as pulmonary hypertension upon SARS-CoV-2 infection.

## Data Availability

The original contributions presented in the study are included in the article/Supplementary Material, further inquiries can be directed to the corresponding author.
